# The long-term immunogenicity of recombinant hepatitis B virus (HBV) vaccine: contribution of universal HBV vaccination in Italy

**DOI:** 10.1186/s12879-015-0874-3

**Published:** 2015-03-25

**Authors:** Nicola Coppola, Anna Rita Corvino, Stefania De Pascalis, Giuseppe Signoriello, Eliana Di Fiore, Albert Nienhaus, Evangelista Sagnelli, Monica Lamberti

**Affiliations:** Department of Mental Health and Public Medicine, Section of Infectious Diseases, Second University of Naples, Via L. Armanni 5, Naples, 80133 Italy; Department of Experimental Medicine, Section of Hygiene, Occupational Medicine and Forensic Medicine, Second University of Naples, Naples, Italy; Department of Mental Health and Public Medicine, Section of Statistic, Second University of Naples, Naples, Italy; Institute for Health Service Research in Dermatology and Nursing (IVDP), Center of Excellence for Epidemiology and Health Service Research for Healthcare Professionals (CVcare), University Medical Center Hamburg-Eppendorf, Hamburg, Germany

**Keywords:** HBV infection, HBV vaccination, Anti-HBs titer, Healthcare students

## Abstract

**Background:**

Universal hepatitis B virus (HBV) vaccination of newborn babies was introduced in Italy in 1991 and was extended to 12-years-old children for the first 12 years of application so as to cover in a dozen years the Italian population aged 0-24 years. The aim of this study was to identify factors associated with long-term immunogenicity against HBV 17 years after primary vaccination in students attending medical schools in Naples, Italy.

**Methods:**

1,704 students attending the school of medicine, schools of the healthcare professions, or postgraduate medical schools of the Second University of Naples, Italy, from September 2012 to December 2013 were enrolled in this study. Of these, 588 had been vaccinated against HBV in infancy and 1,116 when 12 years old. Multivariate logistic regression analysis was used to identify factors associated with the level of long-term immunogenicity.

**Results:**

All vaccinated subjects were HBsAg/anti-HBc negative: 270 (15.8%) had an anti-HBs titer between 1 and 9 IU/L, 987 (57.9%) between 10 and 400 IU/L, and 447 (26.3%) over 400 IU/L. When compared with the latter two subgroups, those with anti-HBs titers lower than 10 IU/L were younger (24 ± 5.2 years vs. 26 ± 4.9 years, *p* < 0.000), more frequently students attending a healthcare school (59% vs. 47%, *p* < 0.001), and more frequently had been vaccinated in infancy (50% vs. 31.5%, *p* < 0.0001). Multivariate logistic regression identified age at vaccination as the only factor independently associated with an anti-HBs titer <10 IU/L (OR: 2.43; C.I. 95%: 1.57–3.76, *p* = 0.001).

**Conclusions:**

Universal HBV vaccination in Italy has been more effective in generating a prolonged protective response in subjects vaccinated at adolescence than in infancy. Students with a low anti-HBs titer should be considered for a booster dose because most will be exposed to the risk of acquiring HBV for decades.

## Background

Infection with hepatitis B virus (HBV) is a leading cause of acute and chronic liver disease worldwide [1]. The World Health Organization (WHO) estimates that, globally, about 2 billion people have been infected with HBV, more than 350 million are chronically infected, and nearly one million per year die from its acute or chronic sequelae, such as fulminant hepatitis, liver cirrhosis, and hepatocellular carcinoma [2]. The prevalence of HBV-related hepatitis varies across countries: in industrialized West European countries and North America, the prevalence of HBV surface antigen (HBsAg) positivity in the general population is less than 2% (low endemicity); in most countries of the Mediterranean, East Europe, and Asia it ranges between 2–8% (intermediate endemicity); whereas it is over 8% in some developing countries in Far-East Asia and Sub-Saharan Africa (high endemicity) [3,4].

In Italy, the epidemiology of HBV infection has changed substantially over the last 50 years: there has been a remarkable, progressive reduction in the incidence of acute hepatitis B (from 10/100,000 inhabitants in 1984 to 0.85/100,000 in 2012) and in the percentage of HBV-related cases among patients with chronic hepatitis (from 60% in 1975 to nearly 8% in 2010) [5,6]; moreover, the prevalence of chronic carriers of HBsAg in the general population has decreased from nearly 3% in the 1980s to 1% or less in 2010 [5]. The reasons for this may be due to a number of relevant events occurring in Italy over the last three decades, including improved socio-economic conditions; a consistent reduction in the number of large families, in which HBV is often transmitted among siblings [7]; the national educational campaigns against HIV infection; mandatory screening for women during pregnancy and/or at the time of delivery; and a mass vaccination campaign against HBV [8,9].

Universal HBV vaccination of newborn babies was introduced in Italy in 1991 and was extended to 12-year-old children for the first 12 years of application so as to cover in a dozen years the Italian population aged 0–24 years. The schedule involves HBV vaccination doses at months 0, 1, and 6, starting from the third month of life for infants. In Italy, HBV vaccination is also recommended for people at risk of acquiring HBV infection [10-12].

A debated issue is how long a protective antibody response may persist after vaccination. A study performed in Italy in 2003 showed that after primary vaccination of infants and adolescents, the antibody response persisted at levels considered protective (>10 IU/L) for at least 10 years in most subjects [13]. However, data on the persistence of the efficacy of vaccination for longer periods are scant and fragmentary [14-18].

The present study was therefore carried out to evaluate the long-term immunogenicity and effectiveness of HBV vaccination and to identify independent predictive factors of long-term immunogenicity. To this end, students attending the medical and healthcare schools of the Second University of Naples, Italy, were enrolled in this study.

## Methods

From September 2012 to December 2013, we anonymously screened the levels of serum HBsAg, anti-HBs, and anti-HBc in all students attending the 6th year of the medical school, the 1st year of the health profession schools (nursing, pediatric nursing, radiology, and midwifery), and the postgraduate medical schools of the Second University of Naples, Naples, Italy. The participation rate for this study was 100%.

A pre-coded questionnaire on demographics, previous exposure to HBV, and HBV vaccination was filled in by each student. Out of the total of 1,727 students examined, 23 had escaped HBV vaccination. The remaining 1,704 had been vaccinated and were enrolled for this study. Of these, 588 had received a course of 3 pediatric doses (10 μg) of recombinant hepatitis B vaccine at their 3rd, 5th, and 11th month of postnatal life and were classified as “vaccinated in infancy” for this paper; 1,116 had received the course of 3 adult doses (20 μg) of the same vaccine when 12 years old. The information on HBV vaccination given by the students was always checked against their vaccination cards.

HBV serum markers (HBsAg, anti-HBs, and total anti-HBc) were determined using commercial immunoenzymatic assays (Abbott Laboratories, North Chicago, IL, USA). Anti-HBs titers were measured on a calibration curve generated with the WHO reference standard, and are expressed in IU/L. In particular, actual values were obtained for anti-HBs titers between 10 and 400 IU/L, and for this interval the geometric mean was calculated with standard procedures; however, for values found <10 IU/L or >400 IU/L, the laboratory readout indicated only “under 10 IU/L” or “over 400 IU/L”, respectively.

The efficacy of anti-HBV vaccination in our cohort was calculated with Orenstain’s equation [19]: VE (%) = [(ARU - ARV)/ARU] x 100, where VE is vaccine efficacy (the per cent reduction in disease incidence attributable to vaccination), ARU is the attack rate in unvaccinated subjects, and ARV is the attack rate in vaccinated subjects.

All procedures were performed in compliance with the Declaration of Helsinki, with the current healthcare standards indicated by the Italian Ministry of Health and in accordance with the guideline established by the Ethic Committee of the Azienda Ospedaliera of the Second University of Naples. The Italian law considers the students attending the medical schools as health care workers since they make their clinical practice in the University Hospitals and it is mandatory for the Universities to control the immunological protection of both health care workers and students against infectious agents like HBV. This is established by the Legislative Decree n. 81, article 279 of the Italian Republic, April 9, 2008. Thus, an approval of an Ethic Committee is not required, but, according to the Italian law for retrospective studies, only a communication, that was done, is required. Moreover, in full agreement with the rationale and the aim of the study, all students signed a written informed consent form. The sera were tested blindly, and all personal information regarding the students were protected according to Italian law.

Statistical analysis was performed with StatGraph, version 3.0. Continuous variables are given as mean ± standard deviation, and categorical variables as the absolute value and relative frequency. Differences in means were evaluated with unpaired Student *t*-test, and the chi-squared test was applied to categorical variables. A *p*-value <0.05 was considered to be statistically significant. Odds ratio (OR), with a 95% confidence interval (CI), was estimated by a logistic regression model to evaluate the confounding between the variation of anti-HBs titers <10 IU/L with sex, age at vaccination, years from vaccination, and the type of school attended as covariates. A *p*-value <0.05 was considered to be statistically significant.

## Results

**Figure 1:**
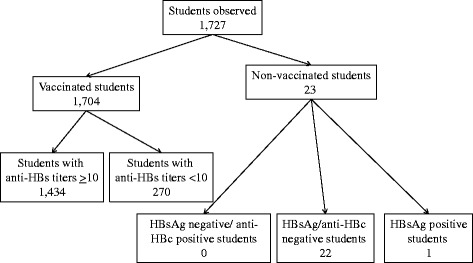
**Flow chart of the students studied at the Second University of Naples from September 2012 to December 2013.**

Of the 23 students who had escaped HBV vaccination, 22 were HBsAg/anti-HBs/anti-HBc negative, and one was HBsAg positive. All vaccinated students were HBsAg/anti-HBc negative (Figure 1). The efficacy of anti-HBV vaccination was thus 100%.

The demographic and epidemiologic characteristics of the 1,704 vaccinated students are given in Table 1. One thousand thirty-three students (60.6%) were female and 1,685 (98.9%) were of a Caucasian Italian ethnic background. The ages of the 525 students attending the school of medicine and the 829 students attending a healthcare profession school were 26.5 ± 3.7 and 23.8 ± 4.9 years, respectively; the 350 postgraduate medical school students were older (29.7 ± 2.2 years). The majority (84.2%) of the students had an anti-HBs titer >10 IU/L: 57.9% between 10 and 400 IU/L and 26.3% over 400 IU/L (Table 1).

**Table 1:** **Demographics of the vaccinated, HBsAg-negative students enrolled**

**Characteristic**		**Value**
N° of subjects		1,704
Age, years		26 ± 4.7
Females		1,033 (60.6)
Years since vaccination		17.7 ± 3.9
Place of birth:	Italy	1,685 (98.9)
	Elsewhere	19 (1.1)
Attending:	School of medicine	525 (30.8)
	Healthcare profession school	829 (48.6)
	Postgraduate medical school	350 (20.6)
Vaccination conducted:	in infancy	588 (34.6)
	at 12 years old	1116 (65.4)
Year of vaccination:	pre-1992	188 (11.0)
	1992–1993	445 (26.1)
	1994–1995	156 (9.2)
	1996–1997	176 (10.3)
	1998–1999	236 (13.8)
	2000–2001	304 (17.8)
	2002	199 (11.7)
HBsAb titer:	<10 IU/L	270 (15.8)
	10–400 IU/L	987 (57.9)
		447 (26.3)
	>400 IU/L	

Continuous variables are given as mean ± SD; categorical variables are given as n° (%).

Compared with students vaccinated at their 12th year of age, those vaccinated in infancy were younger (21.5 ± 0.9 vs. 27.9 ± 4.4, *p* <0.000), more frequently female (72% vs. 55%, *p* <0.000), and more frequently attending a healthcare profession school than either the school of medicine or a postgraduate medical school (94% vs. 24.7%, *p* <0.000) (Table 2). Overall, a longer period of time had elapsed since their vaccination. Moreover, the group of students vaccinated in infancy had a significantly higher prevalence of individuals with a low (<10 IU/L) anti-HBs titer (23.0 vs. 12.1%), more subjects with an anti-HBs titer ranging between 10 and 400 IU/L (67.3 vs. 53%), and a definitely lower rate of subjects with an anti-HBs titer >400 IU/L (9.7 vs. 34.9%), *p* <0.000). The geometric mean of anti-HBs titer (calculated among students with a level ranging 10–400 IU/L) was higher for the group vaccinated in adolescence (59.5 vs. 30.4, *p* <0.000).

**Table 2:** **Characteristics of the 1,704 vaccinated students stratified by age at vaccination**

		**Vaccinated in infancy**	**Vaccinated at 12 years old**	***p***
N° of students		588	1,116	
Age, years		21.5 ± 0.9	27.9 ± 4.4	0.000
Females		421 (72)	613 (55)	0.000
Place of birth:	Italy	584 (99.5)	1101 (99)	n.s.
	Elsewhere	4 (0.5)	15 (1)	
Attending:	School of medicine	33 (6)	492 (44)	0.000*
	Healthcare profession school	555 (94)	274 (24.7)	
	Postgraduate medical school	0	350 (31.3)	
Years from vaccination		21.5 ± 0.9	15.6 ± 3.2	0.000
Students with HBsAb < 10 IU/L		135 (23)	135 (12.1)	0.000
Students with HBsAb 10–400 IU/L		396 (67.3)	591 (53)	0.000
Genometric mean, IU/L		30.4	59.6	0.000**
Students with HBsAb >400 IU/L		57 (9.7)	390 (34.9)	0.000
Students with HBsAb <10 IU/L:	Female	91/421 (21.6)	67/613 (11)	n.s.
	Male	44/167 (26.5)	68/503 (13.5)	
	Vaccinated 1991–1995	135/588 (23)	13/202 (6.4)	0.000
	Vaccinated 1996–2002	-	122/914 (13.3)	-

*Healthcare profession school students vs. all medical school students (graduate + postgraduate).

**Mann–Whitney Test.

Continuous variables are given as mean ± SD; categorical variables are given as n° (%).

To render the time from vaccination comparable in the two groups, a separate evaluation was performed on the sub-group of students vaccinated between 1991 and 1995. This assessment confirmed the above-mentioned findings: in fact, of the 588 students vaccinated in infancy during this period, 23% had an anti-HBs titer lower than 10 IU/L, 67.3% had a titer between 10 and 400 IU/L, and 9.7% had a titer over 400 IU/L; whereas of the 202 vaccinated during adolescence in the same period, the prevalences were 6.4%, 60.4%, and 33.2%, respectively (*p* <0.000). Even for this sub-group, the geometric mean of anti-HBs titer in students with a level ranging 10–400 IU/L was higher in those vaccinated in adolescence (56.5 vs. 30.5 IU/L, p < 0.0001).

The characteristics of the vaccinated students stratified according to HBsAb titer are given in Table 3. The subjects with an anti-HBs titer <10 IU/L were younger, more frequently attending a healthcare profession school, and more frequently had been vaccinated in infancy. Females were prevalent to a similar degree in both groups.

**Table 3:** **Characteristics of students stratified by anti-HBs titer**

		**<10 IU/L**	**≥10 IU/L**	***P***
N° of students		270	1,434	
Age, years		24 ± 5.18	26 ± 4.86	0.000
Females		158 (58.5)	875 (60)	n.s
Attending:	School of medicine	66 (24.4)	459 (32)	0.0001*
	Healthcare profession school	159 (59)	670 (47)	
	Postgraduate medical school	45 (16.6)	305 (21)	
Vaccinated	in infancy	135 (50)	452 (31.0)	0.000
	at 12 years old	135 (50)	982 (67.0)	
Year of vaccination	pre-1992	36 (13.0)	153 (11.0)	n.s
	1992–1993	92 (34.0)	352 (24.4)	
	1994–1995	20 (7.0)	136 (9.6)	
	1996–1997	25 (10.0)	151 (10.5)	
	1998–1999	26 (10.0)	210 (14.5)	
	2000–2001	37 (14.0)	267 (18.5)	
	2002	34 (12.0)	165 (11.5)	
Years from vaccination		18.29 ± 3.97	17.57 ± 3.85	0.005

*Healthcare profession school students vs. all medical school students (graduate + postgraduate). Continuous variables are given as mean ± SD; categorical variables are given as n° (%).

To identify factors independently associated with an anti-HBs titer <10 IU/L, a logistic regression analysis was performed with sex, age at vaccination, type of school attended, and years elapsed from vaccination as the variables (Table 4). The analysis identified age at vaccination as the only independent predictor of low anti-HBs titer (OR: 2.43; C: 95%, 1.57–3.76, *p* =0.00).

**Table 4:** **Logistic regression analysis of variables related to variation of anti-HBs titer <10 IU/L**

**Parameter**	**Odds ratio**	**95% CI**	***p***
Gender: female vs. male	0.77	0.59–1.02	0.07
Age at vaccination: at infancy vs. adolescence	2.43	1.57–3.76	0.00
Type of school: health profession vs. all medical	0.96	0.66–1.40	0.83
Years from vaccination	0.99	0.95–1.04	0.83

OR, Odds Ratio.; 95% CI, 95% confidence interval.

## Discussion

Universal vaccination against HBV infection was introduced in Italy in 1991. All newborn babies were vaccinated (at month 3, 5, and 11) to prevent the risk of acquiring HBV infection by perinatal and familial transmission, while all children aged 12 years were vaccinated to prevent HBV transmission by unsafe sexual activity or intravenous drug addiction. With this strategy, the Italian population aged 0–24 was covered by 2003. It has also allowed us to compare the immunogenicity and efficacy of the HBV vaccine in 588 students vaccinated during infancy and in 1,116 students vaccinated during adolescence; only 23 students in the cohort escaped the universal HBV vaccination program, mostly because of socio-cultural reasons.

HBV vaccination had an excellent efficacy (100%), since none of the vaccinated students have been infected with HBV, as reflected by the absence of positivity to serum HBsAg or anti-HBc after a mean period of nearly 17 years; on the other hand, one (4.3%) of the 23 students who were not vaccinated acquired an HBV infection and became a chronic HBsAg carrier. The high immunogenicity of HBV vaccination is demonstrated by the observation that nearly 90% of students vaccinated during adolescence and three-quarters of those vaccinated at infancy had an anti-HBs titer ≥10 IU/L, which is conventionally accepted as protective. In addition, students with an anti-HBs titer <10 IU/L had a detectable antibody titer ranging 1–9 IU/L. Indeed, there is evidence, confirmed also in the present study, that the protection induced by the recombinant HBV vaccine persists even when the anti-HBs titer declines below 10 IU/L [20-25]. The immunogenicity was higher in students vaccinated during adolescence, as shown by the higher prevalence of students with an anti-HBs titer >400 IU/L, the higher prevalence of those with anti-HBs titers ranging 10–400 IU/L, and by the lower prevalence of subjects with an anti-HBs titer <10 IU/L. Moreover, the geometric mean of the sub-group of subjects with an anti-HBs titer 10–400 IU/L was significantly higher in subjects vaccinated at an the older age. This finding was independent of time elapsed from vaccination to screening, as shown by the sub-group of students vaccinated between 1991 and 1995. Indeed, multivariate analysis identified vaccination at infancy as the only factor associated with a low (<10 IU/L) anti-HBs titer. Thus, vaccination in adolescence results in a more effective HBV immunogenicity, most probably reflecting the progressive improvement of the immune system during childhood [26]. Indeed, the immune system in infancy is characterized by impaired T cell function, lower interaction between B and T cells, restriction of the immunoglobulin repertoire, and low affinity antibody response [26]. These factors, along with the presence of serum anti-HBs in some mothers [27], might affect the response to HBV vaccine in newborn babies and explain the lower immunogenicity of HBV vaccine when administered in infancy. These observations should induce healthcare authorities to reconsider their vaccination strategies in infancy for a more effective response.

As mentioned before, it is common opinion that HBV-vaccinated subjects with an anti-HBs titer that has decreased to below 10 UI/L or that have become anti-HBs negative are still considered protected against HBV infection, since the immunological memory to HBsAg may persist even in these cases and ensure a rapid rise of protective antibodies in the case of an HBV assault [13,18,28]. Students attending healthcare profession schools are considered potentially exposed to hospital infections to a similar degree as healthcare workers (HCWs) [29] and will probably be exposed to HBV for decades once they have become active HCWs themselves. Furthermore, it has been estimated that the incidence of injuries due to sharp objects in HCWs ranges from 1.4 to 9.5 per 100 HCWs per year, and that transmission of HBV results in 0.42 HBV infections per 100 sharp-object injuries per year [30,31]. Therefore, a booster dose should be considered for all subjects with a low anti-HBs titer [32-35].

## Conclusions

To the best of our knowledge, the present study is the first to evaluate the efficacy and immunogenicity of HBV vaccination in a large series of subjects with a high chance of being exposed to HBV for decades*.* HBV vaccination has an excellent efficacy – as demonstrated by the absence of HBsAg and anti-HBc in all vaccinated students – and high immunogenicity – as indicated by the high anti-HBs titers found 17 years after vaccination. However, the effect is stronger when conducted in adolescence. Worldwide, universal HBV vaccination is administrated in infancy, but this practice produces lower immunogenicity than vaccination in adolescence or adulthood. Moreover, although immunological memory may persist for years after HBV vaccination, even in the absence of anti-HBs, the possibility remains that the immune system of HCWs vaccinated in infancy and presenting with a low anti-HBs titer may become overwhelmed by a highly infectious HBV inoculum during a long professional life. Thus, the administration of a booster dose of HBV vaccine should be considered for these individuals.

## Abbreviations

HBV: Hepatitis B virus
WHO: World Health Organization
HBsAg: HBV surface antigen
HCPs: Hospital workers
